# Use of elastic stability analysis to explain the stress-dependent nature of soil strength

**DOI:** 10.1098/rsos.150038

**Published:** 2015-04-22

**Authors:** Kevin J. Hanley, Catherine O'Sullivan, M. Ahmer Wadee, Xin Huang

**Affiliations:** 1 Department of Civil and Environmental Engineering, Imperial College London, London SW7 2AZ, UK; 2 Department of Civil and Environmental Engineering, The University of Hong Kong, Pokfulam Road, Hong Kong, People's Republic of China

**Keywords:** soil mechanics, failure, buckling

## Abstract

The peak and critical state strengths of sands are linearly related to the stress level, just as the frictional resistance to sliding along an interface is related to the normal force. The analogy with frictional sliding has led to the use of a ‘friction angle’ to describe the relationship between strength and stress for soils. The term ‘friction angle’ implies that the underlying mechanism is frictional resistance at the particle contacts. However, experiments and discrete element simulations indicate that the material friction angle is not simply related to the friction angle at the particle contacts. Experiments and particle-scale simulations of model sands have also revealed the presence of strong force chains, aligned with the major principal stress. Buckling of these strong force chains has been proposed as an alternative to the frictional-sliding failure mechanism. Here, using an idealized abstraction of a strong force chain, the resistance is shown to be linearly proportional to the magnitude of the lateral forces supporting the force chain. Considering a triaxial stress state, and drawing an analogy between the lateral forces and the confining pressure in a triaxial test, a linear relationship between stress level and strength is seen to emerge from the failure-by-buckling hypothesis.

## 2. Background

The shear strength of sand is often described using a Mohr–Coulomb linear failure envelope [1,2]. This relates shear strength at failure (*τ*_*ff*_) and the effective normal stress on the failure plane (*σ*′_*ff*_) as

2.1 $$\tau_{\text{ff}}=\sigma_{\text{ff}}^{\prime }\tan\phi^{\prime },$$

where *ϕ*′ is the angle of shearing resistance, sometimes termed the friction angle. Recall that Coulomb friction relates the normal force and maximum shear force along a surface (interface) as

2.2 $$T_{f}=N\tan\phi_{\mathrm{surface}},$$

where *T*_*f*_ is the maximum shear (tangential) force that can be attained along the surface, *N* is the normal force and *ϕ*_surface_ is the friction angle of the interface [3]. The similarity between equations (2.1) and (2.2) gives rise to use of the term ‘friction angle’ in soil mechanics. When triaxial stress conditions are encountered, equation (2.1) is better expressed [4] as

2.3 $$\sigma_{1f}^{\prime }-\sigma_{3f}^{\prime }=(\sigma_{1f}^{\prime }+\sigma_{3f}^{\prime })\sin\phi^{\prime }$$

or

2.4 $$\sigma_{1f}^{\prime }=\sigma_{3f}^{\prime }\left(\frac{1+\sin\phi^{\prime }}{1-\sin\phi^{\prime }}\right),$$

where *σ*′_1*f*_ and *σ*′_3*f*_ are the major and minor principal stresses at the point of failure, respectively. For sands, two angles of shearing resistance are of particular importance: the critical state (*ϕ*′_*cv*_) and the peak (*ϕ*′_*p*_).

The critical state soil mechanics (CSSM) framework, proposed by Roscoe *et al*. [5] and documented by Schofield & Wroth [6], relates the deviator stress (*q*) and mean effective stress (*p*′) at the critical state as

2.5 $$q=Mp^{\prime },$$

where *M* is a critical state parameter. For a triaxial stress state, *q*=*σ*′_1_−*σ*′_3_ and *p*′=(*σ*′_1_+2*σ*′_3_)/2, where *σ*′_1_ is the major principal stress and *σ*′_3_ is both the minor principal stress and the confining pressure. Equation (2.5) can also be expressed as equation (2.6) for a triaxial stress state:

2.6 $$\sigma_{1}^{\prime }=\sigma_{3}^{\prime }\left(\frac{3+2M}{3-M}\right).$$

Equations (2.4) and (2.6), which describe failure according to the Mohr–Coulomb and the CSSM frameworks, respectively, both give a linear relationship between *σ*′_1_ and *σ*′_3_.

Soil strength is typically attributed to originate from both frictional resistance at the contacts between grains and the kinematics of the relative motion of grains [7]. The kinematic constraint is usually related to dilation and the initial state parameter; this is related to the difference between *ϕ*′_*p*_ and *ϕ*′_*cv*_ [8,9]. Countless laboratory experiments (e.g. triaxial tests of Verdugo & Ishihara [10] and plane strain tests of Wanatowski & Chu [11]) have confirmed that, for a given sand with a fixed mineralogy and particle size distribution and hence a constant *ϕ*′ value, *τ*_*ff*_ does increase linearly with stress level. However, the evidence to suggest that friction at the particle contacts is the key factor explaining the increase of *τ*_*ff*_ with increasing *ϕ*′ is not entirely convincing. Mitchell & Soga [7] clearly acknowledged the ample experimental evidence which indicates that *ϕ*′_*cv*_ is not solely a function of mineralogy and highlighted the influence of both particle size distribution and particle shape. Mitchell and Soga proposed that *ϕ*′_*cv*_ is determined by a combination of ‘true friction’ and particle rearrangement including fabric development.

**Figure 1.**
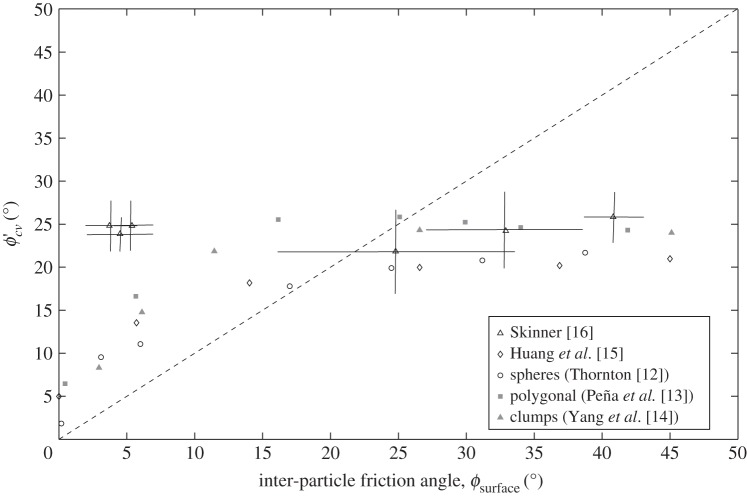
Correlations between critical state angle of shearing resistance (*ϕ*′_*cv*_) and inter-particle friction angle (*ϕ*_surface_), including DEM data from Thornton [12], Peña *et al.* [13], Yang *et al.* [14] and Huang *et al.* [15], with experimental data from Skinner [16].

The discrete element method (DEM) facilitates parametric studies that have examined the influence of particle–particle contact friction on *ϕ*′_*cv*_. The DEM simulations of Thornton [12], Peña *et al.* [13], Yang *et al.* [14] and Huang *et al.* [15], among others, have shown that *ϕ*′_*cv*_ is sensitive to the inter-particle friction angle (*ϕ*_surface_) only at low values of *ϕ*_surface_, as illustrated in figure 1. These numerical results indicate that frictional sliding along the interfaces defined by particle contacts is not the key mechanism determining the critical state strength findings. These numerical studies are in agreement with the earlier experimental observations of Skinner [16], which are also presented in figure 1.

**Figure 2.**
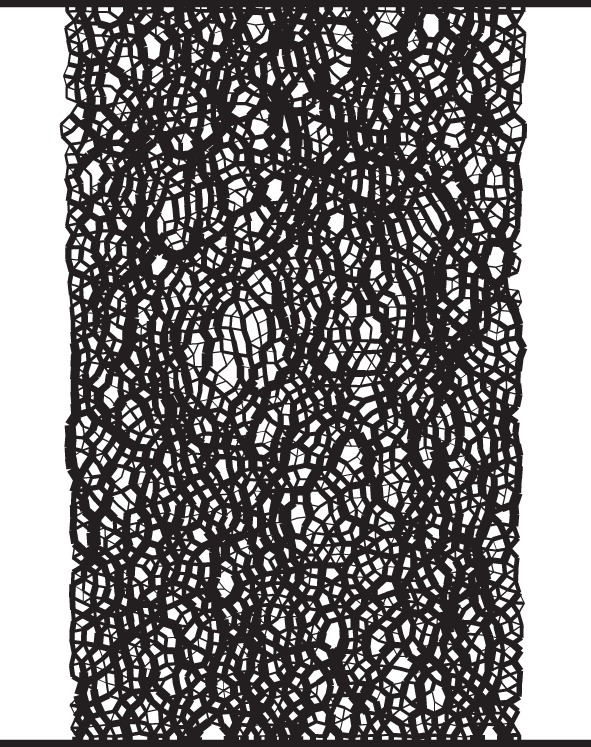
Contact force network in two-dimensional DEM biaxial test simulation of 2376 discs at an axial strain of 5.5%. The thicknesses of the lines joining the centres of contacting disks are proportional to the magnitude of the contact force.

It is now broadly accepted that the stress distribution within a sand is heterogeneous; discrete subnetworks or chains of contacting relatively highly stressed particles form upon loading. These ‘strong force chains’ are aligned with the major principal stress orientation and they have been observed in DEM simulations [17], in photoelastic experiments using analogue sands [18], and in real sands using both image analysis of photographs [19] and micro-computed tomography [20]. Figure 2 illustrates these force chains using data from a two-dimensional DEM simulation of biaxial compression. There are clear hypotheses in the literature linking soil failure to the buckling or collapse of these strong force chains [21]. At the critical state, or in a shear band, it seems that there is continuous formation of new force chains concurrent with failure of pre-existing force chains by buckling [22].

Mitchell & Soga [7] explicitly attribute the non-proportional relationship between *ϕ*_surface_ and *ϕ*′_*cv*_ illustrated in figure 1 to the formation of the strong force network, and state that friction (i.e. *ϕ*_surface_) acts to stabilize this network but is not ‘the direct source of macroscopic resistance to shear’. Mitchell and Soga's statement is really speculation that force chain buckling may provide a valid conceptual framework for soil failure; proof of this hypothesis requires a rational explanation for the linear stress–strength relationship expressed in equations (2.1), (2.4) and (2.6). Here, following the earlier contributions of Hunt *et al.* [23] and O'Sullivan *et al.* [24], several simple models of isolated force chains are used to examine the link between buckling and stress-dependent strength. In each of these models, the particles are represented by nodes that are connected by rigid links to model the strong force chain and lateral forces are applied to represent the weak force network orthogonal to *σ*′_1_ that transmits the confining pressure (i.e. *σ*′_3_). The relationship between the lateral forces, which simulate *σ*′_3_, and the post-buckling load following collapse of the force chain, which is analogous to *σ*′_1_, is assessed. The main difference between this work and the earlier work of Hunt *et al.* [23] is the development of an explicit relationship between the principal stresses within a real sample subjected to triaxial loading and the output of these analogue models. In the prior contribution of O'Sullivan *et al.* [24], data obtained from DEM simulations of true triaxial tests were used as input for a spring-and-link model to show that the relative support offered to strong force chains in the minor and intermediate principal stress directions explains the observed dependency of sample strength on the intermediate stress ratio, $b=(\sigma_{2}^{\prime }-\sigma_{3}^{\prime })/(\sigma_{1}^{\prime }-\sigma_{3}^{\prime })$. This paper considers the influence of confining pressure on the sample strength without relying on data from either simulations or experiments to generate the model.

First, a two-dimensional model containing a single node is developed (§3). This model is sufficiently simple to permit an analytical solution for the post-critical equilibrium path to be obtained in closed form without resorting to numerical bifurcation analysis. This model is then extended into three dimensions and multiple nodes (§4).

## 3. Development of a two-dimensional single-node model

This study neglects explicit consideration of the supporting role played by inter-particle friction to increase the stability of strong force chains and considers only the contribution of the lateral supports. Previously, Tordesillas *et al.* [22] acknowledged that lateral supporting contacts play a key role in supporting the force chains. Using DEM, Barreto & O'Sullivan [25] showed that both inter-particle friction and the weaker network of contacts oriented orthogonal to the major principal stress direction contribute to the stability of these force chains.

**Figure 3.**
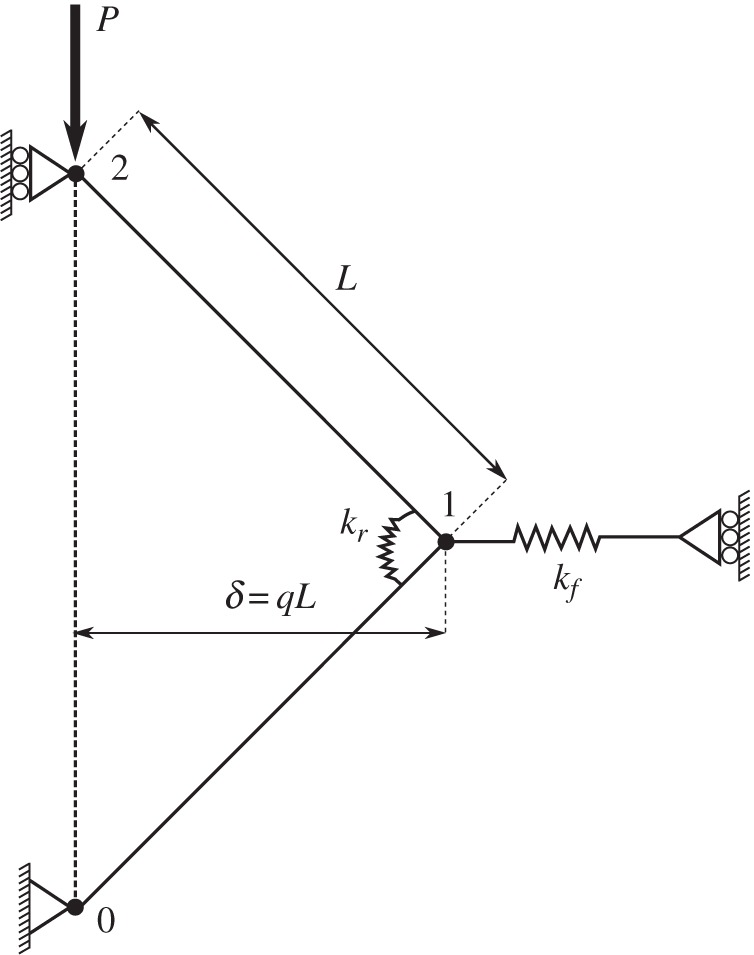
Two-dimensional spring-and-link model with one node and a linear spring of stiffness *k*_*f*_ at a deflection of *δ*, based on the two-dimensional model of Hunt *et al.* [23].

The analogue models used in this paper have a similar form to the three-dimensional model developed by O'Sullivan *et al.* [24], which was based on a two-dimensional model proposed by Hunt *et al.* [23]. Consider the very simple abstraction of a force chain shown in figure 3 which contains one node and two rigid links of equal length *L*. Two springs, a lateral spring of stiffness *k*_*f*_ and a rotational spring of stiffness *k*_*r*_, resist motion of the node from its equilibrium position at which both springs are unstressed. The lateral spring represents the lateral force chains or the weak contact network oriented in the direction of *σ*′_3_ that acts to support the strong force chains. The rotational spring represents the rotational resistance present at the contacts owing to particle geometry effects and friction.

O'Sullivan *et al.* [24] considered a true triaxial stress state when developing a three-dimensional spring-and-link model, i.e. *σ*′_3_≤*σ*′_2_≤*σ*′_1_, and the relative values of the spring stiffnesses were chosen by considering the axial stiffnesses in the *σ*′_3_ and *σ*′_2_ directions measured in DEM simulations of true triaxial tests [26]. In this paper, the earlier work of O'Sullivan *et al.* has been advanced, so that a constant lateral confining force (*F*_L_) can be applied to mimic a constant value of *σ*′_3_, i.e. the lateral support is force-controlled rather than stiffness-controlled.

**Figure 4.**
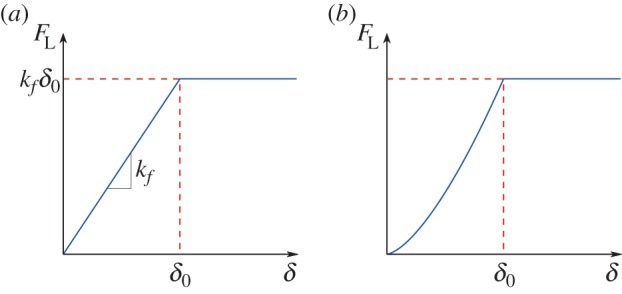
Increase of lateral confining force with deflection according to a ramp function with (*a*) a linear spring or (*b*) a Hertzian spring.

Two different types of lateral spring were used: a linear spring for which the force is directly proportional to deflection (i.e. *F*_L_=*k*_*f*_*δ*) and a nonlinear Hertzian spring for which *F*_L_=*K*_*f*_*δ*^3/2^. Here, *k*_*f*_ corresponds to the stiffness of the linear spring with SI units of N m^−1^, whereas *K*_*f*_ is the stiffness coefficient of the Hertzian spring (N m^−3/2^). For both types of spring, the force increases until a deflection *δ*_0_ is attained; thereafter, the force becomes constant and independent of compressive deflection of the spring, *δ*, as shown in figure 4. It is necessary to have a piecewise description to avoid singularities at the critical state.

Referring to figure 3, the bottom link is pin-jointed, and a load *P* is applied to the uppermost link, increasing from zero until buckling occurs. A total potential energy function, *V* , is developed for the system, which has the same form as that described by O'Sullivan *et al.* [24], i.e.

3.1 $$V=U_{L}+U_{R}-P\Delta ,$$

where *U*_L_ and *U*_L_ represent the total elastic potential energy of the lateral and rotational springs, respectively, and *P*Δ is the work done by the load *P*. The *U*_L_ term was developed by dividing the response into two parts. The strain energy stored in the linear lateral spring, i.e. the area enclosed by figure 4*a*, is

3.2 $$\left.\begin{array}{rlr} U_{L} & =\frac{1}{2}k_{f}\delta^{2} & \;\text{if }\delta \leq \delta_{0}\\ \;\text{and}\;\;U_{L} & =\frac{1}{2}k_{f}\delta_{0}^{2}+k_{f}\delta_{0}(\delta -\delta_{0}) & \;\text{if }\delta >\delta_{0} \end{array}\right\}$$

while the corresponding equations for the strain energy in the Hertzian spring, figure 4*b*, are

3.3 $$\left.\begin{array}{rlr} U_{L} & =\frac{2}{5}K_{f}\delta^{5/2} & \;\text{if }\delta \leq \delta_{0}\\ \;\text{and}\;\;U_{L} & =\frac{2}{5}K_{f}\delta_{0}^{5/2}+K_{f}\delta_{0}^{3/2}(\delta -\delta_{0}) & \;\text{if }\delta >\delta_{0}. \end{array}\right\}$$

Of course, this assumes that *δ*>0 during the loading history. Following the approach of O'Sullivan *et al.* [24], *P*Δ and *U*_L_ can be expressed as

3.4 $$P\Delta =2PL(1-\sqrt{1-q^{2}})$$

and

3.5 $$U_{R}=2k_{r}{\left(\arcsin q\right)}^{2}=2k_{r}q^{2}\text{ to leading order.}$$

Equations (3.4) and (3.5) can be substituted into equation (3.1) along with the appropriate expressions for *U*_L_ and *δ*=*qL*. At equilibrium, the potential energy is stationary, i.e. energy dissipation due to friction or particle breakage is neglected and so d*V*/d*q*=0. Thus, solving for *P* for the four distinct cases discussed gives the following relationships

3.6 $$\left.\begin{array}{rlr} P & =\sqrt{1-q^{2}}\left(\frac{2k_{r}}{L}+\frac{k_{f}L}{2}\right) & \;\text{if spring is linear and }\delta \leq \delta_{0}\\ P & =\sqrt{1-q^{2}}\left(\frac{2k_{r}}{L}+\frac{k_{f}\delta_{0}}{2q}\right) & \;\text{if spring is linear and }\delta >\delta_{0}\\ P & =\sqrt{1-q^{2}}\left(\frac{2k_{r}}{L}+\frac{K_{f}q^{1/2}L^{3/2}}{2}\right) & \;\text{if spring is Hertzian and }\delta \leq \delta_{0}\\ \;\text{and}\;\;P & =\sqrt{1-q^{2}}\left(\frac{2k_{r}}{L}+\frac{K_{f}\delta_{0}^{3/2}}{2q}\right) & \;\text{if spring is Hertzian and }\delta >\delta_{0}. \end{array}\right\}$$

Any load beyond the transition point (which occurs at *δ*=*δ*_0_ in this simple model) can be identified as a suitable resistance because it is beyond that point at which the constant force confinement becomes valid. This post-buckling load represents the vertical load that maintains equilibrium; for a given displacement, *δ*, any load greater than this resistance would cause collapse. Note that it is essential to compare the model at the same deflection. For the single-node model, this was arbitrarily chosen as *q*=0.003, because this value is always higher than *δ*_0_/*L*.

## 4. Extension to a three-dimensional multiple-node model

**Figure 5.**
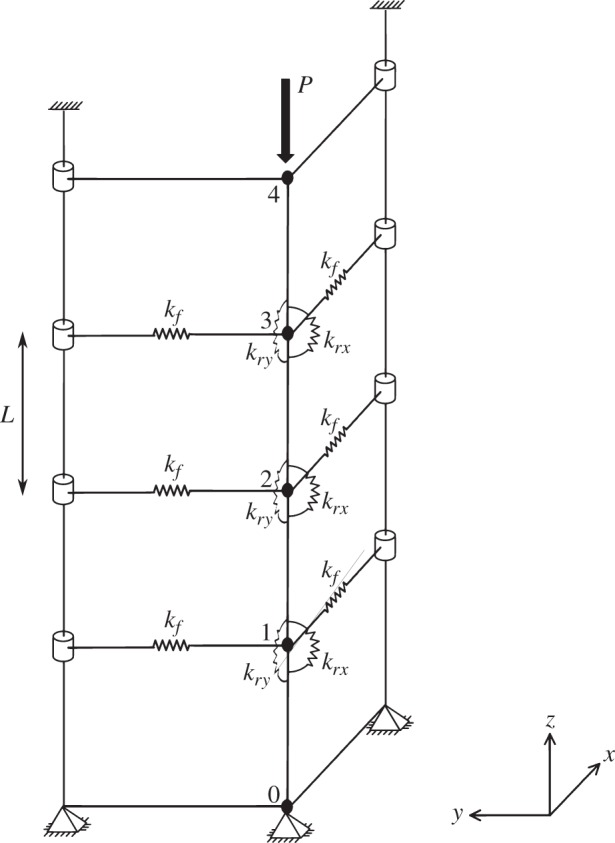
Three-dimensional spring-and-link model with three nodes in the undeflected state with unstressed lateral and rotational springs (Adapted from O'Sullivan *et al.* [24]).

The two-dimensional single-node model developed in §3 can be extended to three dimensions and multiple nodes. A vertical force chain is idealized as *N* nodes connected by *N*+1 rigid links, each of length *L*, as shown in figure 5 for the *N*=3 case. The displacements of any node *i* in the *x* and *y* directions from the undeflected state with unstressed springs are given by *q*_*ix*_*L* and *q*_*iy*_*L*, respectively. Each node is supported by four springs: two linear lateral springs of equal stiffness (*k*_*f*_) and two rotational springs.

One of the rotational spring stiffnesses, *k*_*ry*_, was specified by fixing the ratio *k*_*ry*_:*k*_*rx*_ at 1.1 to avoid numerical difficulties with concurrent zero eigenvalues arising during the matrix inversion required for the solution that is outlined below. Two approaches were adopted to estimate suitable values of *k*_*rx*_. One approach was to set the rotational stiffnesses at arbitrarily chosen constant values. Alternatively, the stiffnesses were taken to be proportional to the load *P*; this is comparable to some of the rolling resistance models proposed in the literature where the rolling stiffness is proportional to the normal contact force [22,27–29].

The relevant equations to describe both *U*_L_, the total elastic potential energy of the rotational springs, and *P*Δ, the work done by the load *P*, were developed by O'Sullivan *et al.* [24]:

4.1 $$\begin{array}{rl} U_{R} & =\frac{1}{2}k_{rx}\sum_{i=1}^{N}{\left(\arcsin(q_{yi+1}-q_{yi})-\arcsin(q_{yi}-q_{yi-1})\right)}^{2}\\ & \;+\frac{1}{2}k_{ry}\sum_{i=1}^{N}{\left(\arcsin(q_{xi+1}-q_{xi})-\arcsin(q_{xi}-q_{xi-1})\right)}^{2} \end{array}$$

and

4.2 $$P\Delta =P\sum_{i=0}^{N}\Delta_{i}=PL\sum_{i=0}^{N}\left(1-\sqrt{1-{\left(\sqrt{q_{xi+1}^{2}+q_{yi+1}^{2}}-\sqrt{q_{xi}^{2}+q_{yi}^{2}}\right)}^{2}}\right).$$

For the three-dimensional multiple-node model, linear lateral springs were used which have the force–deflection characteristics shown in figure 4*a*. For this multiple-node model, negative deflections are permitted for which a symmetric relationship between *F*_L_ and *δ* is assumed, i.e. *F*_L_=−*k*_*f*_|*δ*_0_| if *δ*≤−|*δ*_0_|. The deflections of the two lateral springs are

4.3 $$\delta_{ix}=L\left(1-\sqrt{q_{yi}^{2}+{\left(1-q_{xi}\right)}^{2}}\right)$$

and

4.4 $$\delta_{iy}=L\left(1-\sqrt{q_{xi}^{2}+{\left(1-q_{yi}\right)}^{2}}\right).$$

The *U*_L_ term was developed by dividing the response into two parts for each node *i*. When *δ*_*ix*_ or *δ*_*iy*_≤*δ*_0_, the strain energy stored in the lateral spring is $\frac{1}{2}k_{f}\delta_{ix}^{2}$ or $\frac{1}{2}k_{f}\delta_{iy}^{2}$, respectively, as in O'Sullivan *et al.* [24]. When *δ*_*ix*_ or *δ*_*iy*_>*δ*_0_, the respective strain energy terms become

4.5 $$U_{L}=\frac{1}{2}k_{f}\delta_{0}^{2}+k_{f}\delta_{0}(\delta_{ix}-\delta_{0})$$

or

4.6 $$U_{L}=\frac{1}{2}k_{f}\delta_{0}^{2}+k_{f}\delta_{0}(\delta_{iy}-\delta_{0}).$$

The total strain energy stored in the 2*N* linear springs is obtained by summing the energy terms for both lateral springs over all *N* nodes.

The expression for the total potential energy function, *V* , contains five constants: *k*_*f*_, *k*_*rx*_, *k*_*ry*_, *L* and *δ*_0_; *V* can be non-dimensionalized as $\tilde{V}$ by dividing all terms by *k*_*f*_*L*^2^ to leave three dimensionless groups of constants:

4.7 $${\tilde{\delta }}_{0}=\frac{\delta_{0}}{L},\;{\tilde{k}}_{rx}=\frac{k_{rx}}{k_{f}L^{2}}\;\mathrm{and}\;{\tilde{k}}_{ry}=\frac{k_{ry}}{k_{f}L^{2}}.$$

By non-dimensionalizing, the work done by the load becomes

4.8 $$\tilde{P}\tilde{\Delta }=\tilde{P}\sum_{i=0}^{N}\left(1-\sqrt{1-{\left(\sqrt{q_{xi+1}^{2}+q_{yi+1}^{2}}-\sqrt{q_{xi}^{2}+q_{yi}^{2}}\right)}^{2}}\right),$$

where $\tilde{P}=P/(k_{f}L)$. As for the two-dimensional single-node model, the potential energy is stationary at equilibrium, i.e.

4.9 $$\frac{\partial \tilde{V}}{\partial q_{xi}}=\frac{\partial \tilde{V}}{\partial q_{yi}}=0,$$

where *i*=1,2,…,*N*. These partial derivatives were calculated symbolically using Maple 17.00 [30]. The CodeGeneration package within Maple was used to generate a Fortran equations file suitable for input to AUTO-07p [31] following the approach of O'Sullivan *et al.* [24].

For each model evaluation, non-dimensional resistances (${\tilde{P}}_{f}$) were identified on the equilibrium path at the same Euclidian norm of the deflections. An arbitrary value was chosen which is much larger than all values of ${\tilde{\delta }}_{0}$ adopted. This procedure is similar to picking post-buckling loads at *q*=0.003 for the two-dimensional single-node model. ${\tilde{P}}_{f}$ is taken to be analogous to the principal stress *σ*′_1_. Thus, any pattern in the variation of ${\tilde{P}}_{f}$ with *F*_L_ is analogous to a variation in *σ*′_1_ with *σ*′_3_. In the majority of the simulations, the initial displacements of all nodes were zero (i.e. *q*_*xi*_=*q*_*yi*_=0 for all *i*). As real materials contain flaws, an imperfection was included in a subset of the simulations by giving the middle node an initial perturbation of equal size in both the *x* and *y* directions. The simulations were run using two different numbers of nodes: three and seven. The confining force was varied by using a range of values for ${\tilde{\delta }}_{0}$.

## 5. Results and discussion

### 5.1 Two-dimensional single-node model

Four separate cases were considered for the two-dimensional model: both linear and Hertzian lateral springs were used, and for each spring type, the confining force *F*_L_ was varied by adjusting either the spring cut-off, *δ*_0_=*q*_0_*L*, or the spring stiffness parameter (i.e. *k*_*f*_ or *K*_*f*_ for linear or Hertzian springs, respectively). It is noted that the confining force at *δ*>*δ*_0_ is linearly proportional to the spring stiffness parameter for both linear and Hertzian lateral springs (i.e. *F*_L_=*k*_*f*_*δ* or *K*_*f*_*δ*^3/2^), whereas *F*_L_ is nonlinearly related to deflection for the Hertzian model.

**Figure 6.**
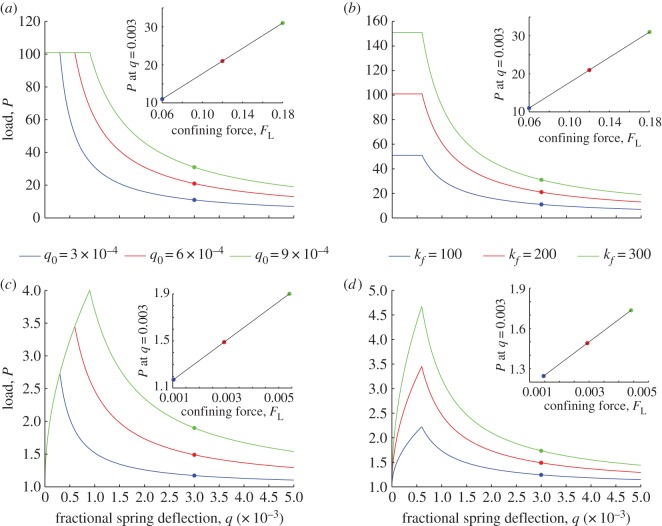
Variation of vertical load with fractional spring deflection for four cases of the two-dimensional single-node model. Panels (*a*,*b*) use a linear lateral spring, whereas (*c*,*d*) use a Hertzian spring. For cases (*a*,*c*), the confining force was varied by changing *q*_0_ at constant stiffness, whereas for (*b*,*d*), the stiffness was varied at constant *q*_0_. The smaller inset plots show the load at *q*=0.003 versus confining force.

Figure 6 shows model evaluations for the four cases considered. For those two cases in which *δ*_0_ was varied, *q*_0_ values of 3×10^−4^, 6×10^−4^ or 9×10^−4^ were used with a fixed *k*_*f*_ of 200 N m^−1^ (linear springs) or 200 N m^−3/2^ (Hertzian springs). In the other two cases, a fixed *q*_0_ of 6×10^−4^ was used with *k*_*f*_ or *K*_*f*_ values of 100, 200 or 300 (N m^−1^ or N m^−3/2^). Regardless of spring type or the level of the confining force, there is a clear transition point at *q*=*q*_0_ characterized by a sharp change of slope beyond which the load *P* decreases monotonically with increasing deflection. The loads may be compared at any arbitrarily chosen deflection along these decreasing paths; the small inset subfigures plot load against confining force at *q*=0.003. The load is linearly related to confining force even if the initial lateral spring behaviour is nonlinear (i.e. Hertzian) and this linear relationship holds for all *q* values that exceed *q*_0_. The latter point is important as soil is inherently a nonlinear material. Because the column resistance and confining force in this simple model represent *σ*′_1_ and *σ*′_3_, a linear relationship emerges between *σ*′_1_ and *σ*′_3_, irrespective of the manner in which the confining force is applied, i.e. equations (2.4) and (2.6) emerge from this simple abstraction.

### 5.2 Three-dimensional multiple-node model

The two-dimensional single-node model is convenient for analysis because of its simplicity. However, real force chains exist in three dimensions and can comprise many contacting particles. For this reason, it is necessary to confirm that the linear relationship between confining force and resistance observed for the two-dimensional single-node model is also true for the three-dimensional multiple-node model developed in §4.

**Figure 7.**
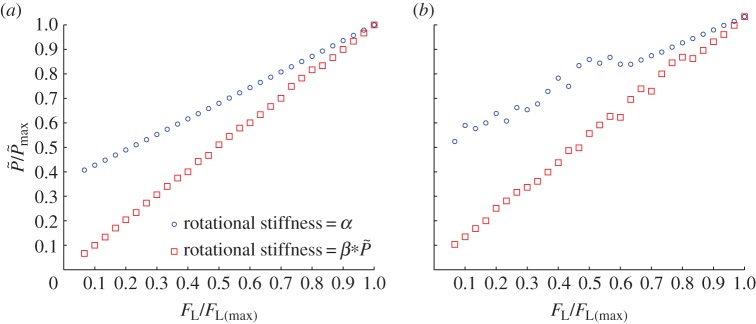
Variation of post-buckling force, quantified at the same deflection for each model evaluation, with confining force for three-dimensional (*a*) three- and (*b*) seven-node models in which both forces have been non-dimensionalized and scaled by their respective maximum values. Both constant rotational stiffness and rotational stiffnesses which vary linearly with load have been used.

Figure 7*a* shows that the column resistance increases linearly with confining force for a three-node chain using two definitions of ${\tilde{k}}_{rx}$: ${\tilde{k}}_{rx}=\alpha$ or ${\tilde{k}}_{rx}=\beta \tilde{P}$, where *α* and *β* are arbitrarily chosen constants. Figure 7*b* is similar to figure 7*a* except that a chain length of seven nodes is considered. Because the aim of this study is to give semi-qualitative trends, the non-dimensional confining and post-buckling forces for each set of multiple-node simulations were normalized by their respective maximum values.

The critical buckling force increases linearly with confining force for both chain lengths, regardless of whether ${\tilde{k}}_{rx}$ is maintained constant or ${\tilde{k}}_{rx}\propto \tilde{P}$. The small deviations from linearity in figure 7*b* are due to the inability of AUTO to find the lowest eigenvalue in a limited number of cases owing to close proximity with other eigenmodes causing numerical problems, as discussed by Wadee *et al.* [32]. Because the post-buckling force is taken to be analogous to *σ*′_1_ and the confining force is analogous to *σ*′_3_, the trends expressed in equations (2.4) and (2.6) are again captured by this model which does not contain an inter-particle friction parameter. It is noted that when the rotational stiffness is independent of confining force, the rotational springs are sufficient to prevent buckling at negligibly small forces. Hence, $\tilde{P}>0$ in the absence of a confining load for both cases of constant rotational stiffness. When ${\tilde{k}}_{rx}\propto \tilde{P}$, the contribution of the rotational springs to resist buckling is initially zero, so buckling can take place at tiny values of $\tilde{P}$.

**Figure 8.**
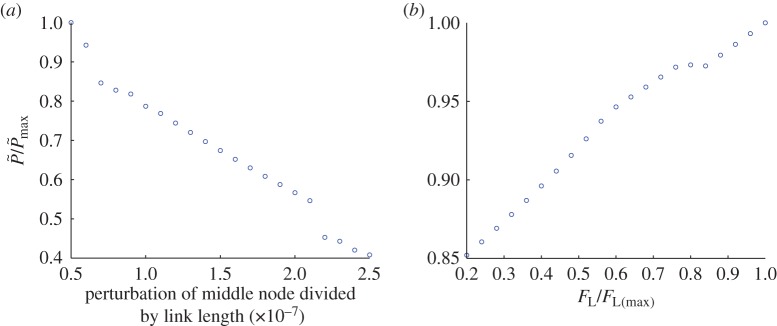
(*a*) Decrease in resistance with increase in size of a perturbation applied to the middle node of a three-node column when all other model inputs are held fixed; (*b*) variation of post-buckling force with confining force for a three-node model containing an initial perturbation of 10^−7^ of link length (both forces have been non-dimensionalized and scaled by their respective maximum values).

Figure 7 is for a column in which the nodes are initially collinear (vertical). In a real material, the particles comprising the force chains are not perfectly aligned (as illustrated in figure 2). The three-node model was modified by giving the middle node a small initial perturbation. Figure 8*a* shows the sharp reduction of ${\tilde{P}}_{f}$ that occurs as the size of this initial perturbation increases while all other model inputs are held constant. Although the perturbations remain small, ${\tilde{P}}_{f}$ decreases substantially as the perturbation size is increased. This sensitivity to perturbations is expected from elastic buckling theory, but inhibits the development of a quantitative relationship between a force chain in a real sand (or in a discrete element simulation representing a real sand) and the simple abstract models considered here. Figure 8*b* shows that when the force chain is initially perturbed, in this case by 0.00001% of link length, the linear relationship between resistance and confining force observed in figure 7 still fundamentally exists.

## 6. Conclusion

The dependency of soil strength upon stress level is well known and it is commonly described as a frictional relationship. Prior experiments and DEM simulations have shown that *ϕ*′_*p*_ and *ϕ*′_*cv*_, which are often referred to as friction angles, are not simply related to particle surface friction, *ϕ*_surface_, suggesting that a framework which considers the critical state strength to be a purely frictional strength has a tenuous scientific basis. There is by now ample evidence to indicate that, when subject to a non-isotropic stress state, sand particles and their contacts are oriented in the direction of *σ*′_1_ to form columns of preferentially stressed particles. Abstractions of these columns can be created using simple spring-and-link models. By using these abstract models and controlling the applied lateral forces, it has been shown that the actual post-buckling strength of these columns is approximately linearly related to the lateral supporting force when the confining force is basically constant. This model, therefore, indicates that the linear relationships between *τ*_*ff*_ and *σ*′_*f*_ and between *q* and *p*′ emerge from the fundamental mechanics of force chain buckling. The model explains the existence of these relationships despite the absence of an explicit link between inter-particle friction (*ϕ*_surface_) and the critical state angle of shearing resistance (*ϕ*′_*cv*_).

## Data accessibility

All of the data used to plot figures 7 and 8 are deposited in Dryad. Sets of AUTO input files for these simulations (*.f and c.*) and the associated AUTO output files (fort.7, fort.8, fort.9) are also available at http://dx.doi.org/10.5061/dryad.05478.
